# Pre-ischemic exercise alleviates oxidative damage following ischemic stroke in rats

**DOI:** 10.3892/etm.2014.1874

**Published:** 2014-07-31

**Authors:** RUI FENG, MIN ZHANG, XIAO WANG, WEN-BIN LI, SHI-QING REN, FENG ZHANG

**Affiliations:** 1 Department of Neurology, The Third Hospital of Hebei Medical University, Shijiazhuang, Hebei 050051, P.R. China; 2 Department of Pathophysiology, Hebei Medical University, Shijiazhuang, Hebei 050017, P.R. China; 3 Department of Rehabilitation Medicine, The Third Hospital of Hebei Medical University, Shijiazhuang, Hebei 050051, P.R. China; 4 Hebei Provincial Orthopedic Biomechanics Key Laboratory, The Third Hospital of Hebei Medical University, Shijiazhuang, Hebei 050051, P.R. China

**Keywords:** pre-ischemic exercise, oxidative damage, ischemic stroke, malondialdehyde, superoxide dismutase

## Abstract

Physical exercise has been proved to be neuroprotective in clinical trials and animal experiments. However, the exact mechanism underlying this neuroprotective effect remains unclear. The aim of the present study was to explore whether pre-ischemic treadmill training could act as a form of ischemic preconditioning in a rat following ischemic stroke by reducing oxidative damage. Fifty-four rats were randomly divided into three groups (n=18 per group): Sham surgery, middle cerebral artery occlusion (MCAO) without exercise and MCAO with exercise. Subsequent to treadmill training, ischemic stroke was induced by occluding the MCA for 1.5 h, followed by reperfusion. Six rats in each group were evaluated for neurological deficits and then sacrificed by decapitation to calculate the infarct volume. The remaining rats in each group were sacrificed to detect the level of superoxide dismutase (SOD) activity (n=6) and malondialdehyde (MDA) concentration (n=6). The results indicated that pre-ischemic exercise training reduced brain infarct volume and neurological deficits, increased SOD activity and decreased the concentration of MDA following ischemic stroke. In conclusion, treadmill exercise training prior to MCAO/reperfusion increased the antioxidant ability and decreased the oxidative damage in the brain subsequent to ischemic stroke.

## Introduction

In the past two decades, animal experiments have indicated the protective effects of physical exercise on ischemic stroke, including enhanced survival rates, a reduction in neurological deficits, an alleviation of blood-brain barrier (BBB) dysfunction and an improvement in neurovascular integrity (1–5). Therefore, exercise preconditioning has begun to attract increasing attention.

Our previous review summarized the association between exercise preconditioning and brain ischemic tolerance, involving a series of pathological changes following ischemic stroke (6). However according to the search results at that time, there was no study on the antioxidative effect of pre-ischemic exercise following cerebral ischemia.

Regular exercise training has been shown to downregulate levels of free radicals (7), thus reducing the peroxidation levels of lipids or proteins in the brain of the rat (8). Furthermore, regular exercise training promoted the effect of antioxidant enzymes, including superoxide dismutase (SOD) and glutathione peroxidase (9–11). Since malondialdehyde (MDA) plays a key role in the process of ischemic stroke, the concentration of MDA in brain tissues and plasma has been used to assess the severity of neuronal ischemic injury (12–14).

A recent study indicated that pre-ischemic treadmill training for three weeks could alleviate brain oxidative damage by suppressing 4-hydroxy-2-nonenal-modified proteins and 8-hydroxy-2′-deoxyguanosine following ischemic stroke (15). In the study, the SOD activity in the exercise plus sham group was significantly higher than that in the sham group. However, whether pre-ischemic exercise can regulate the ischemic stroke-induced changes in SOD activity and MDA level remains unknown. Thus, the aim of the present study was to explore whether exercise preconditioning can regulate SOD and MDA and thereby decrease oxidative stress following MCAO.

## Materials and methods

### Animals

Fifty-four male Sprague Dawley rats, each weighing 200–220 g, were obtained from the Hebei Province Laboratory Animal Center (Shijiazhuang, China). All the rats were kept under a 12-h light/dark cycle. Food and water were available *ad libitum*. All the procedures in this study were approved by the Animal Care and Use Committee of Hebei Medical University (Shijiazhuang, China).

### Treadmill training

Rats were randomly divided into three groups (n=18 per group): Sham surgery, middle cerebral artery occlusion (MCAO) without exercise and MCAO with exercise. Prior to formal training, rats in the MCAO with exercise group underwent adaptive running exercise training for two days at a speed of 5–8 m/min for 30 min/day on a treadmill training machine (DSPT-202 Type 5-Lane Treadmill; Litai Biotechnology Co., Ltd., Shishi, China). Following the adaptive exercise training, the rats underwent formal treadmill training at a speed of 20 m/min, 30 min/day for six days every week. The rats in the sham and MCAO without exercise groups did not receive exercise but were allowed to run freely in their living cages during the same time period.

### MCAO model

Following the treadmill training, rats received MCAO surgery. Animals were anesthetized using 4% chloral hydrate (10 ml/kg, intraperitoneal) and were administered further doses if necessary to maintain the anesthesia state during the process of surgery. The body temperature of the rat was maintained at 37°C by a heating pad. The surgical procedures were performed in accordance with those described by Longa *et al* (16) with minor modification.

Briefly, the left external carotid artery (ECA), common carotid artery (CCA) and internal carotid artery (ICA) were firstly exposed. A monofilament (4–0 nylon suture) with a blunted poly-L-lysine coated tip (Beijing Sunbio Biotech Co., Ltd., Beijing, China.) was lightly inserted into the ECA. The suture then moved through the CCA and ICA, and finally occluded the MCA at its origin. After 90 min of MCAO, reperfusion was performed by removing the filament.

For the sham group, the CCA, ECA and ICA underwent the same procedures without occlusion of the MCA. The associated physiological parameters were monitored by a Blood Gas and Electrolyte System (ABL505; Radiometer Medical ApS, Copenhagen, Denmark). Rats were assessed 24 h after reperfusion according to a widely accepted scale as follows: 0, no neurological symptoms; 1, unable to completely extend the front jaw on the contralateral side; 2, rotating while crawling and falling to the contralateral side; 3, unable to walk without assistance; and 4, unconsciousness (16).

### Determination of brain infarct volume

Twenty-four hours after reperfusion, animals were sacrificed by decapitation under chloral hydrate (10%) anesthesia. The whole brains were stored in a refrigerator at −20°C for 10 min, following which each brain was cut into six coronal sections (2 mm thick) between the anterior pole and the optic chiasm in the center. All tissues were immediately infiltrated into a 2% 2,3,5-triphenyltetrazolium chloride solution at 37°C thermostat for 30 min, then fixed in 4% paraformaldehyde buffer. After 24 h, a digital camera (DC240; Kodak, Rochester, NY, USA) and imaging software (Adobe Photoshop 7.0; Adobe Systems Inc., San Jose, CA, USA) were used to capture images and calculate the infarction area. The total infarction volume was equal to the sum of the infarct area in each section. In order to minimize the error caused by brain edema, the corrected formula to calculate infarct volume was as follows: Infarct volume = contralateral hemisphere region - non-infarcted region in the ipsilateral hemisphere. The following formula was used to calculate infarct percentage: Infarct percentage = infarct volume/volume of the contralateral hemisphere × 100%

### Preparation of brain tissue

Twenty-four hours after reperfusion, the left hemisphere of the rat brain was harvested following perfusion transcardially with 0.9% saline under deep anesthesia. Briefly, the tissues were immersed into radio-immunoprecipitation assay lysis buffer and homogenized mechanically at 37°C. The homogenate was centrifuged at 10,000 × g for 4 min at 4°C and the supernatant was collected. Protein concentration was measured using an Enhanced Bicinchoninic Acid Protein Assay kit (Beyotime Institute of Biotechnology, Haimen, China).

### SOD activity detection

SOD activity in the brain tissue was detected using the methodology described by Oyanagui (17) according to the kit instructions (Nanjing Jiancheng Bioengineering Institute, Nanjing, China). The main principle was as follows: Superoxide anions were generated in the xanthine and xanthine oxidase system. These superoxide anions oxidized hydroxylamine, resulting in the formation of nitrite. This nitrite reacted with sulfanilic acid and naphthalene diamine, generating a colored product (18). SOD in the brain tissue decreased the superoxide anion concentration, thus reducing the colorimetric signal and absorbance, which was generally measured at 550 nm. One unit of SOD activity was defined as the amount of enzyme that was required to inhibit the 50% reduction of nitroblue tetrazolium in the specified conditions. The procedures were repeated three times in order to obtain the mean values.

### MDA assay detection

The concentration of MDA was detected by a thiobarbituric acid reaction method (19). Thiobarbituric acid reacts with MDA in acidic medium resulting in a pink-colored pigment at 95°C. The absorbance at a wavelength of 532 nm was detected by a microplate reader (DU640; Beckman Coulter Inc., Miami, FL, USA). The procedures were performed based on the kit instructions (Nanjing Jiancheng Bioengineering Institute). MDA content (nmol/mg protein) was calculated using the following formula: Absorbance of sample tube/absorbance of standard tube × 2.5. The procedures were repeated three times in order to obtain the mean values.

### Statistical analysis

Statistical analysis was performed by SPSS for Windows, version 15.0 (SPSS Inc., Chicago, IL, USA). The neurological deficit scores and infarct volume between ischemic rats with and without pre-ischemic exercise were compared by an independent Student’s t-test. The differences in SOD enzyme activity units and MDA concentration among the three groups were analyzed by one-way analysis of variance followed by a post hoc least significant difference test. Data are presented as the mean ± standard deviation. P<0.05 was considered to indicate a statistically significant difference in all statistical assessments.

## Results

### Physiological variables

Fifty-four rats were divided into three groups (n=18 per group): Sham surgery, MCAO without exercise and MCAO with exercise. No significant differences were observed in the partial pressure of oxygen in arterial blood, the partial pressure of carbon dioxide in arterial blood or pH (hydrogen ion concentration) values among the three groups (P>0.05; data not shown).

### Behavioral scores

**Figure 1:**
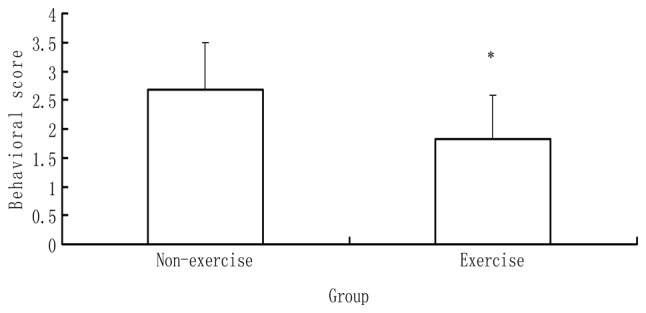
Behavioral scores of the MCAO with and without exercise groups. Data are presented as the mean ± standard deviation (n=6). The behavioral scores for the sham group were all zero (data not shown). ^*^P<0.05 vs. the MCAO without exercise group. MCAO, middle cerebral artery occlusion.

Rats in the three groups were evaluated 24 h after reperfusion. In the sham surgery group, the rats exhibited no neurological symptoms. By contrast, a significant difference in behavioral scores was identified between the MCAO with and without exercise groups (P<0.05), as shown in Fig. 1. The rats in the MCAO with exercise group showed fewer neurological deficits than those in the MCAO without exercise group.

### Infarct volume

**Figure 2:**
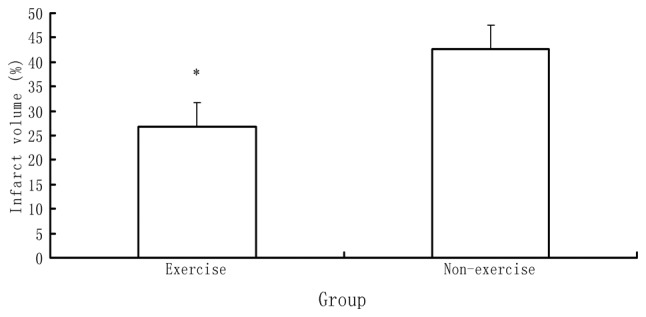
Difference in infarct volume between the MCAO with and without exercise groups. Data are presented as the mean ± standard deviation. The infarct volume of the sham surgery group of six rats was zero (data not shown). ^*^P<0.05 vs. the MCAO without exercise group. MCAO, middle cerebral artery occlusion.

Subsequent to behavioral evaluation, six rats in each group were sacrificed by decapitation to determine the infarct volume. The rats in the sham surgery group showed no ischemic areas. By contrast, a significant difference in infarct volume was identified between the MCAO with and without exercise groups (P<0.05), as indicated in Fig. 2. The rats in the MCAO with exercise group showed a significantly reduced ischemic area in the brain relative to those in the MCAO without exercise group.

### SOD activity

**Figure 3:**
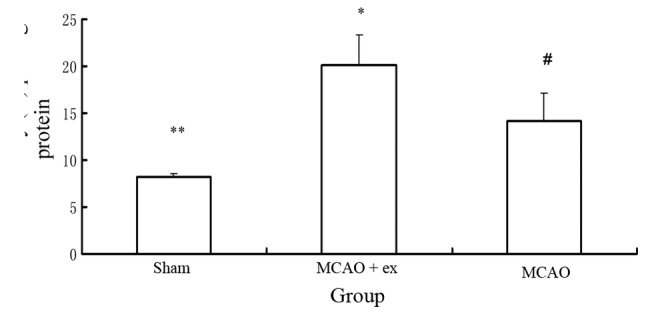
Difference in SOD activity among the three groups. The SOD activity of the MCAO with exercise group was higher than that of the MCAO without exercise group. The MCAO with and without exercise groups showed a higher SOD activity than the sham surgery group. Data are presented as the mean ± standard deviation. ^#^P<0.05 vs. the sham surgery group; ^**^P<0.05 vs. the MCAO with exercise group; ^*^P<0.05 vs. the MCAO without exercise group. MCAO, middle cerebral artery occlusion; MCAO + ex, MCAO with exercise; SOD, superoxide dismutase.

Six rats in each group were sacrificed by decapitation to determine the SOD activity. A significant difference in SOD activity was identified among the three groups (P<0.05), as indicated in Fig. 3. The rats in the MCAO with exercise group showed a higher SOD activity in the brain relative to those in the MCAO without exercise group (P<0.05). The rats in the MCAO with and without exercise groups showed higher SOD activity in the brain relative to those in the sham surgery group (P<0.05).

### MDA concentration

**Figure 4:**
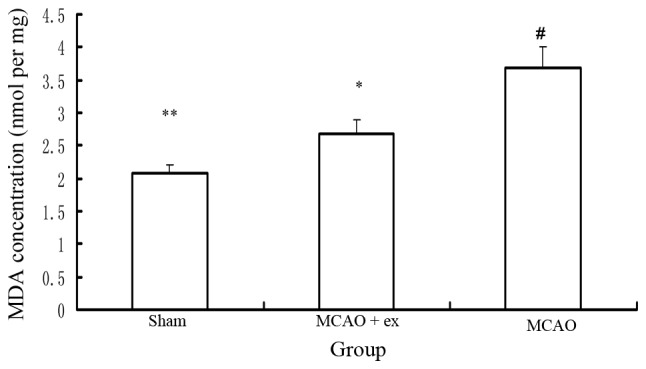
Difference in MDA concentration among the three groups. The MDA concentration of the MCAO with exercise group was lower than that of the MCAO without exercise group. The MCAO with and without exercise groups showed a higher MDA concentration than the sham surgery group. Data are presented as the mean ± standard deviation. ^#^P<0.05 vs. the sham surgery group; ^**^P<0.05 vs. the MCAO with exercise group; ^*^P<0.05 vs. the MCAO without exercise group. MCAO, middle cerebral artery occlusion; MCAO + ex, MCAO with exercise; MDA, malondialdehyde.

Six rats in each group were sacrificed by decapitation to determine the MDA concentration. A significant difference in MDA concentration was identified among the three groups (P<0.05), as indicated in Fig. 4. The rats in the MCAO with exercise group exhibited a lower MDA concentration in the brain relative to those in the MCAO without exercise group (P<0.05). The rats in the MCAO with and without exercise groups showed a higher MDA concentration in the brain relative to those in the sham surgery group (P<0.05).

## Discussion

Ischemic stroke is the third leading cause of mortality in Western countries (20). Therefore, strategies to prevent stroke and reduce brain damage following stroke have attracted increasing attention. A number of interventions have been investigated to achieve this, including physical activity (21).

A series of clinical studies on human subjects have explored the association between pre-ischemic exercise and the risk of ischemic stroke. These studies have produced different results. Certain studies have demonstrated that pre-ischemic exercise results in improved functional outcomes following stroke and a reduction in the ischemic stroke risk (21–24). The mechanisms underlying the neuroprotective effects of exercise pretreatment on post-stroke function and the occurrence of stroke are not clear according to these epidemiological studies, but have been revealed to be associated with the effects of pre-ischemic exercise on metabolic pathways, blood pressure, blood cholesterol and glucose level (22–25). By contrast, a prospective clinical study has indicated that pre-ischemic exercise may reduce the occurrence of ischemic stroke, but does not alleviate dysfunction following ischemic stroke (25). Thus, on the basis of the above-mentioned clinical studies, it is perhaps difficult to conclude that pre-ischemic exercise provides beneficial effects on the pathogenesis of stroke; however, it appears that the majority of the associated evidence demonstrates that pre-ischemic exercise exerts a beneficial effect on both the occurrence of and functional outcomes following ischemic stroke.

With regard to the mechanism underlying the effect of preconditioning exercise following ischemic stroke, animal experiments reported that pre-ischemic exercise improved BBB function and enhanced basal lamina integrity subsequent to ischemic stroke (26). Additionally, pre-ischemic exercise for three weeks increased cerebrovascular integrity in the striatum of rats (27,28). Our previous studies demonstrated that exercise preconditioning reduced the over-release of glutamate and influenced glutamate receptor changes, alleviating brain damage subsequent to stroke (29,30).

All the above-mentioned neuroprotective effects of pre-ischemic exercise may be indirectly associated with the antioxidant ability of exercise pretreatment following ischemic stroke. It is logically speculated that pre-ischemic exercise may alleviate BBB dysfunction, reduce glutamate over-release and increase cerebrovascular integrity so as to reduce oxidative stress following cerebral ischemia.

Lipid peroxidation is induced by high levels of free radicals, which can be caused by the cerebral ischemia-reperfusion (31–33). Long-term exercise training increases the antioxidant abilities of brain tissue (34). The present data also demonstrated that pre-ischemic exercise increased SOD activity and decreased MDA levels, thus providing a protective effect on oxidative injury following MCAO. The pre-ischemic exercise also decreased infarct volume and neurological deficits. However, further study is required to explore the mechanism underlying the antioxidant effect of pre-ischemic exercise training following ischemic stroke.

In conclusion, the results in the present study indicated that treadmill training exercise prior to MCAO/reperfusion increased antioxidant ability and decreased oxidative damage in the brain. Therefore, the pre-ischemic exercise alleviated brain damage and reduced motor dysfunction subsequent to MCAO. These results could be beneficial for the development of rational programs of prevention and treatment for ischemic stroke.
